# Contribution of Fecal Calprotectin and Fecal Immunochemical Tests to the Evaluation of Patients with Ulcerative Colitis

**DOI:** 10.5152/tjg.2024.23510

**Published:** 2024-06-01

**Authors:** Aysun Yakut

**Affiliations:** Department of Gastroenterology, Sefaköy Health Application and Research Center, İstanbul Medipol University, İstanbul, Türkiye

Dear Editor,

I read with interest the article by Hu et al^1^ titled “Evaluation of Mucosal Healing in Ulcerative Colitis by Fecal Calprotectin vs. Fecal Immunochemical Test: A Systematic Review and Meta-analysis,” published on July 11, 2023. As emphasized by Hu et al,^1^ mucosal healing (MH) in patients with ulcerative colitis (UC) is very valuable for the clinician. The clinician wants to use tests that are easy to apply, cheap, practical, accessible, and have high sensitivity and specificity in patient follow-up.^2^ In addition, with the help of simple and practical tests, the clinician wants to monitor remission in UC patients, prevent long-term complications, proactively predict the treatment, change the treatment if necessary, and prevent complications that may develop.^2^ However, as we all know, fecal calprotectin (FC) and fecal immunochemical tests (FIT) are not diagnostic, and their sensitivity and specificity are controversial. Also, there are no clear threshold values. Many gastrointestinal conditions, such as IBD, cholecystitis, diverticulitis, malignancy, infections, nonsteroidal anti-inflammatory drug enteropathy, celiac disease, and microscopic colitis, can cause an increase in FC value.^3^ Again, FIT is mainly known as a screening method for colon malignancies.^4^

Therefore, as Hu et al^1^ said, despite their various limitations, using these tests in patient follow-up is very beneficial to us. However, as a result of the study, Hu et al^1^ emphasized that non-invasive UC biomarkers such as FC and FIT have high diagnostic performance, are reliable, and can replace invasive endoscopy in predicting UC in some patients. As a reader, it is not a direct diagnostic test but an indirect test used in follow-up. Since Hu et al’s^1^ study is a meta-analysis, important differences between the studied populations and the populations for which the recommendation is targeted (true negative (TN), true positive (TP), false negative (FN), and false positive (FP)), unexplained important differences between studies, clinical variability in UC patients with moderate-to-severe activity, and the threshold values of follow-up and non-invasive tests cannot be clearly stated. As the authors note, these uncertainties may contribute to publication bias.

Some disagreements limit the use of non-invasive tests like FC and FIT. Firstly, biomarkers such as FC and FIT are not specific to UC activity. They may be affected by other inflammatory diseases as well as concurrent systemic diseases. They may be elevated in some infectious gastroenteritis, drug-induced colitis, and other inflammatory diseases of the intestine.^5^ Again, in individuals with moderate to severe UC, it may not be appropriate to proceed with aggressive treatment based on disease activity based on FIT and FC values alone. The strategy of using these biomarkers instead of endoscopy should be based on a multidisciplinary evaluation of the appropriate patient. Detection and monitoring of dysplasia in UC patients with high disease age is only possible with endoscopy.^5^ Similarly, in severe UC patients, especially those resistant to corticosteroids, endoscopic evaluation may be required to exclude cytomegalovirus colitis.^5^ In addition, clinical, laboratory, endoscopic, and histopathological remission must be achieved in UC patients before we can call it complete remission. Biomarkers may be insufficient to predict endoscopic and/or histopathological remission.^5^ They may be especially insufficient to distinguish mild activity in UC from histopathological complete remission. On the other hand, patients with UC have mucosal involvement patterns such as proctitis, proctosigmoiditis, and pancolitis. Stool biomarkers may not be sensitive enough to detect inflammation, depending on the segment involved.^5^ Another issue is that the optimal threshold value of biomarkers such as FC and FIT is not known and may differ depending on the cost and risk of predictable interventions.^5^ Unfortunately, tests such as FC and FIT cannot be used interchangeably. To compare a patient’s results over time, the same test should be used. FC and/or FIT values in a single patient may vary during the day. Therefore, confidence in a single measurement may be limited.^5^ Finally, the situation that limits the use of non-invasive tests is that there are interindividual differences in the elevation of biomarkers in patients with intestinal inflammation. Biomarkers may correlate poorly with endoscopic activity. The overall performance and confidence in the use of biomarkers for treatment decisions in a given patient may be higher when these biomarkers are observed longitudinally to correlate with the patient’s endoscopic disease activity (both active disease and remission).^5^

In conclusion, Hu et al^1^ presented a very useful meta-analysis revealing the uses and limitations of FIT and FC, which we frequently use in UC follow-up. Although these tests are not sensitive and specific enough to replace endoscopy, this meta-analysis emphasized that they are easy to use, practical, and inexpensive tests in the follow-up of UC patients, and it was essential to contribute to the literature.
